# Multiplexed promoterless gene expression with CRISPReader

**DOI:** 10.1186/s13059-019-1712-5

**Published:** 2019-06-03

**Authors:** Hengji Zhan, Qun Zhou, Qunjun Gao, Jianfa Li, Weiren Huang, Yuchen Liu

**Affiliations:** 1 Key Laboratory of Medical Reprogramming Technology, Shenzhen Second People’s Hospital, The First Affiliated Hospital of Shenzhen University, Shenzhen, 518035 China; 2 Guangdong Key Laboratory of Systems Biology and Synthetic Biology for Urogenital Tumors, Institute of Translational Medicine, Shenzhen Second People’s Hospital, The First Affiliated Hospital of Shenzhen University, Shenzhen, 518035 China

**Keywords:** CRISPR-Cas9, Promoterless gene expression, AAV vector

## Abstract

**Background:**

Genes are comprised of DNA codes and contain promoters and other control elements for reading these codes. The rapid development of clustered regularly interspaced short palindromic repeats (CRISPR) technology has made possible the construction of a novel code-reading system with low dependency on the native control elements.

**Results:**

We develop CRISPReader, a technology for controlling promoterless gene expression in a robust fashion. We demonstrate that this tool is highly efficient in controlling transcription and translation initiation of a targeted transgene. A notable feature of CRISPReader is the ability to “read” the open reading frames of a cluster of gene without traditional regulatory elements or other cofactors. In particular, we use this strategy to construct an all-in-one AAV-CRISPR-Cas9 system by removing promoter-like elements from the expression cassette to resolve the existing AAV packaging size problem. The compact AAV-CRISPR-Cas9 is also more efficient in transactivation, DNA cleavage, and gene editing than the dual-AAV vector encoding two separate Cas9 elements, shown by targeting both reporter and endogenous genes in vitro and in vivo.

**Conclusions:**

CRISPReader represents a novel approach for gene regulation that enables minimal gene constructs to be expressed and can be used in potential biomedical applications.

**Electronic supplementary material:**

The online version of this article (10.1186/s13059-019-1712-5) contains supplementary material, which is available to authorized users.

## Introduction

DNA code reading is essential to the living cell. This process includes transcription and translation. RNA polymerase transcribes an mRNA copy from DNA, and ribosomes then attach to and translate the mRNA to produce a protein [1, 2]. The genetic code stored in DNA is “interpreted” by gene expression, and such processes can be modulated by promoters, 5′ untranslated regions (UTRs) and other genetic components [3]. These elements usually have a key role in determining the levels of gene expression, and they control cellular behaviors and phenotypes of all organisms [4, 5]. However, native regulatory elements have various types of motifs that are seemingly non-conserved among genes, thus leading to more complex and unpredictable networks. The restructuring of the gene expression pattern by artificial devices represents an important step toward the intentional design and construction of living systems.

Gene therapy is rapidly becoming a new option in multiple fields of medicine. However, it has been limited by challenges in several fundamental areas. For example, it is necessary to express a transgene in a controlled and efficient manner without the use of complex regulatory elements. The current technologies have both advantages and disadvantages, making it difficult to resolve this problem. Inducible promoters allow the expression of a gene to be tuned in a controllable manner, whereas constitutive promoters control the steady and stable expression of a gene [6]. Moreover, multiple large regulatory elements must be used for the simultaneous regulation of expression of several genes [7]. Based on its possible applications, a large gene cassette is not compatible with the limitations of transgene delivery technology. A well-known problem involves the delivery system for CRISPR-Cas9 [8]. Adeno-associated virus (AAV) is currently the most potentially useful gene therapy vector for clinical applications [9], and CRISPR technology is the most valuable gene regulation tool. Unfortunately, the DNA loading capacity of AAV vectors is less than the length of the CRISPR system [10, 11], because AAV cannot package more than ~ 5.0 kb of DNA. In previous studies, the 4.2 kb Cas9 from *Streptococcus pyogenes* (SpCas9) was split and packaged into two separate AAVs, which allowed functional reconstitution of full-length SpCas9 in cells [12–14]. However, this dual-AAV system reduced the efficiency of delivery. Deleting some functional sequences from the Cas9 gene may also reduce its activity.

We have recently reported that the CRISPR system offers a general platform for robust and precise targeting of genes for transcription [15, 16]. In another initial study [17], we reported a modular RNA activator containing the aptamer for eukaryotic initiation factor 4G (eIF4G) that activated target mRNA translation in a 5′ UTR–independent manner. We hypothesized that the ability to bring together a CRISPR transcription factor and a RNA translation activator would enable promoter-independent control of gene expression, offering a new possibility for producing a single AAV containing all CRISPR/Cas9 components. To test this possibility, we developed CRISPReader, a technology that controls gene expression at both the transcriptional and translational levels.

## Results

### CRISPR-based transcriptional factors drive promoterless *Rluc* transcription

To demonstrate that CRISPR dSpCas9-VP64 was capable of supporting transcription in the absence of a common promoter region, a single guide RNA (sgRNA) targeting region (20 bp) was inserted upstream of a minimal TATA box (consensus TATATAA). The coding sequence for *Renilla* luciferase (*Rluc*) was placed downstream of the TATA box, and Rluc therefore served as a reporter for gene expression (Additional file 1: Figure S1a). We reasoned that dCas9-VP64 would bind to the sgRNA targeting region, and the VP64 transcriptional activation domain would then help initiate transcription of the downstream *Rluc* gene (Additional file 1: Figure S1a). To test the ability to activate transcription, human embryonic kidney 293T (HEK-293T) cells were co-transfected with plasmids encoding dCas9-VP64-sgRNA and the *Rluc* reporter. The cytomegalovirus (CMV) promoter–driven *Rluc* and SV40 promoter–driven *Rluc* vectors were also used as positive controls. After 48 h, the luciferase activity was measured, and the results indicated that the dCas9-VP64 system induced a 30-fold increase in luciferase activity compared to cells co-treated with the dCas9-VP64-sgRNA control and the *Rluc* reporter (Additional file 1: Figure S1b). Higher levels of *Rluc* expression (up to 100-fold activation with the CMV promoter) were achieved by the traditional strong CMV and SV40 promoters (Additional file 1: Figure S1b). These results confirmed that the CRISPR transcriptional activation system was useful for inducing transgene expression with a minimal TATA box.

### CRISPReader drives promoterless gene expression

**Fig. 1:**
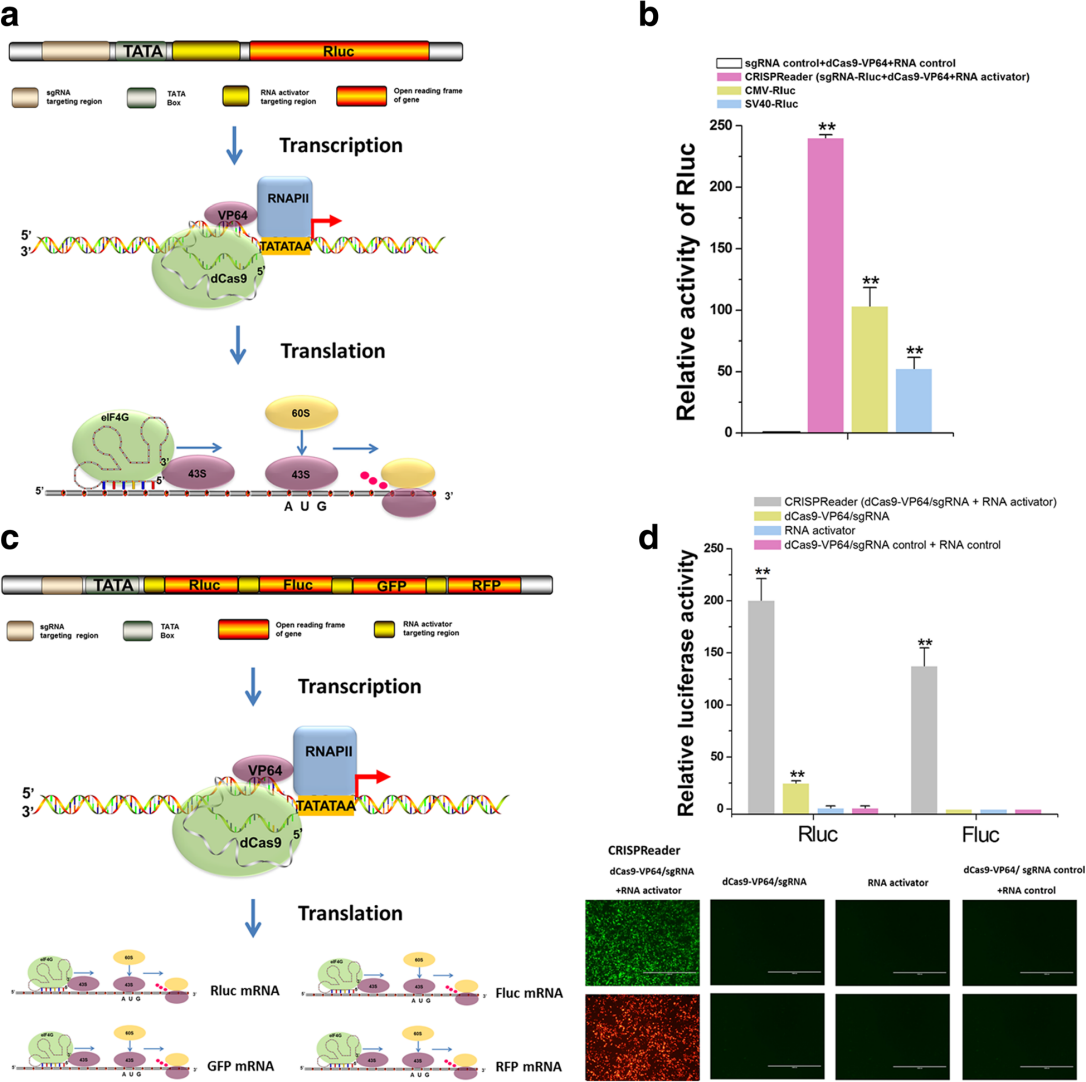
CRISPReader drives promoterless gene expression by coupling the transcriptional and translational mechanisms. **a** CRISPReader was constructed by combining transcriptional and translational platforms. The dCas9-VP64 protein robustly activated transcription of reporter constructs when combined with sgRNA targeting sequences near the TATA box. Then, the RNA activator led to the formation of initiation factor complexes involving eIF4G and recruited ribosomes to initiate translation. **b** The results of the dual luciferase assay. An unregulated TK promoter-driven gene encoding firefly luciferase was used as a control. Reported data are the mean ± SD from five experiments. **P < 0.01, compared with the sgRNA-negative control using the paired, one-sided *t* test. **c** Mechanisms of the CRISPReader designed to drive the gene cluster expression. After dCas9-VP64-mediated transcription, the RNA activators bound to each targeted mRNA and independently initiated mRNA translation. **d** CRISPReader activated the expression of each ORF. The results of the dual luciferase assay are shown at the top. Reported values are presented as the mean ± SD, and the experiments were repeated five times. **P < 0.01, compared with the negative control using the paired, one-sided *t* test. The expressions of GFP and RFP were detected by fluorescent microscopy. Representative images of the transfected cells are shown at the bottom. Scale bar 1000 μm

To further increase the expression level of the promoterless gene, we coupled the transcriptional and translational mechanisms by combining the dCas9-VP64 system with the RNA activator as the CRISPReader module. The RNA activator binds to the 5′ end of *Rluc* mRNA (20 nt) after its transcription and recruits a small ribosomal subunit, resulting in enhanced translational initiation (Fig. 1a). HEK-293T cells were transfected with either the CRISPReader module or the controls. CRISPReader induced a 240-fold increase in luciferase activity compared to the negative control (dCas9-VP64 + sgRNA nonspecific control + RNA activator nonspecific control) (Fig. 1b). Similar results as described in above were achieved with the CMV and SV40 promoters (Fig. 1b). To demonstrate the specificity of the RNA activator, we generated variant RNA activators harboring Watson-Crick transversion mismatches at positions 1–20 (numbered 1–20 in the 3′ to 5′ direction) and tested whether these various RNA activators directed CRISPReader-mediated *Rluc* activation in HEK-293T cells. The results suggested that the RNA activator was very sensitive to a mismatch at any position (Additional file 1: Figure S2). We also tried to explore the regulatory factors that influence the activity of CRISPReader in the context of the *Rluc* construct. To accomplish this objective, we either inserted multiple sgRNA targeting regions upstream of the TATA box or placed multiple RNA activator targeting regions before the initial ATG codon of the *Rluc* ORF. Higher levels of *Rluc* expression were achieved by increasing the number of targeting regions for sgRNA (Additional file 1: Figure S3a and b) or RNA activator (Additional file 1: Figure S4a and b). Together, the results showed that CRISPReader promoted the transcription and translation of a transgene in a robust fashion.

To demonstrate the wide applicability of the RNA activator-based approach for translational regulation, we compared the translation activation efficiencies between the RNA activator and the internal ribosome entry site (IRES), as IRESs are often used for translation initiation in a cap-independent manner [18]. The RNA activator generated a comparable gene activation level to that of the IRES element (Additional file 1: Figure S5a and b). This observation motivated us to investigate whether CRISPReader facilitated the expression of multiple gene ORFs from a single construct in promoter/5′ UTR–independent manner. We constructed a polycistronic reporter vector containing the ORFs of four well-known reporter genes (*Rluc*, *Fluc*, *GFP*, and *RFP*). An RNA activator targeting region (20 nt) was located between each pair of reporter ORFs (Fig. 1c). The transcription of these ORFs was under the control of dCas9-VP64. Translation of the first reporter ORF was initiated in a cap-dependent manner and enhanced by the RNA activator, while translation initiation of other ORFs was only directed by each RNA activator (Fig. 1c). To test this system, we transfected HEK-293T cells with the CRISPReader and the polycistronic reporter construct. We observed simultaneous activation of *Rluc* and *Fluc* by the CRISPReader using the luciferase reporter assay (Fig. 1d). Importantly, activations were mediated by the CRISPReader for two other reporter gene ORFs (*GFP* and *RFP*), as shown by microscope observations. As expected, a single dCas9-VP64 or RNA activator was ineffective for mediating reporter expression except for *Rluc* (Fig. 1d). Thus, this platform could, in principle, be used for reading multiple gene ORFs and constructing a compact gene expression cassette by deleting the promoter-like elements.

### Rational design of a compact AAV-CRISPR-Cas9 with CRISPReader

**Fig. 2:**
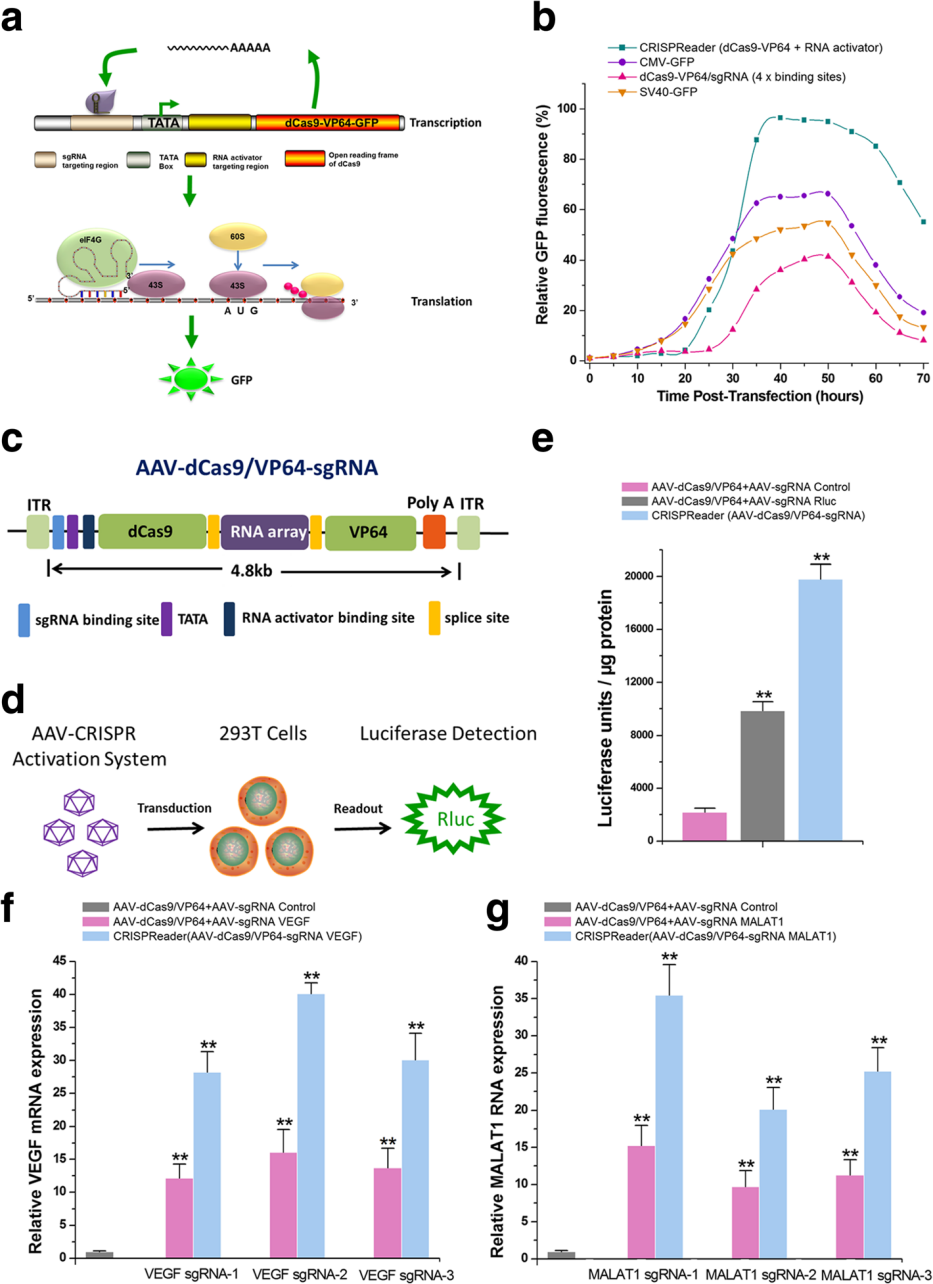
Rational design of a compact AAV-CRISPR-Cas9 system with CRISPReader. **a** Design of a positive feedback loop for expressing dCas9-VP64. The loop amplified the cellular dCas9-VP64-GFP signals generated from the basal transcription of the TATA box. **b** The relative GFP fluorescence at various time points was measured by FACS analysis. **c** Design and construction of the all-in-one AAV-dCas9 system. The Cas9/intron-RNA array gene is shown. **d** Schematic of the mammalian luciferase reporter system used to evaluate transactivation efficiency of the AAV-dCas9 system. **e** The results of the luciferase assay. Data are the mean ± SD from five experiments. **P < 0.01, compared with the negative control using the paired, one-sided *t* test. **f**, **g** The relative RNA levels of VEGF and MALAT1. Data are the mean ± SD from five experiments. **P < 0.01, compared with the negative control using the paired, one-sided *t* test

We then determined whether CRISPReader could be used to downsize the CRISPR expression cassette to bypass the AAV payload limit. We assembled a CRISPR-positive feedback expression loop consisting of the following three steps (Fig. 2a): (1) the initial expression of dCas9-VP64 was achieved by the basal transcriptional activity of the TATA box, (2) the binding of dCas9-VP64 to the sgRNA targeting region upstream of the TATA box promoted its own transcription, and (3) the translation of mRNA was further enhanced by the RNA activator. Using these three steps, dCas9-VP64 expression could be amplified by itself. We constructed a fusion gene of dCas9-VP64 and *GFP* and detected whether the positive feedback loop was successfully constructed by observing GFP reporter expression. HEK-293T cells were transfected with either the CRISPReader construct or the positive controls, and GFP was monitored until 70 h post-transfection. We performed a time course measurement of feedback loop-mediated regulation of *GFP* (Fig. 2b). Our data indicated that the CMV or SV40 promoter could quickly drive *GFP* expression. After a delay of 20 h, the fluorescence signal in cells treated with the CRISPReader construct started to increase very quickly and reached a much higher level than those treated with the CMV or SV40 promoter–driven constructs. A small change in *GFP* expression was observed in the control group, in which a single dCas9-VP64 construct was used to constitute the positive feedback loop. Even the dCas9-VP64 construct with 4 sgRNA binding sites also induced a much slower and weaker response in *GFP* expression when compared to other constructs (Fig. 2b).

After that, we constructed an all-in-one AAV delivery vector for the dSpCas9-VP64 system using the positive feedback loop mediated by the CRISPReader (Fig. 2c). We first deleted the CMV promoter (553 bp) that was responsible for Cas9 expression in previously established systems, and replaced it with the sgRNA binding site, TATA box, and RNA activator binding site. We combined a sgRNA coding sequence with an RNA activator coding sequence to assemble an RNA array and inserted ribozyme sequences at both ends of each designed RNA. Our previous study showed that the primary transcripts could be self-cleaved by the ribozyme to generate the desired RNA molecules in cells [19]. To further reduce the size of the expression cassette of this RNA array, we used a previously reported construct in which the RNA array cassette was inserted into an artificial intron within the coding region of the dCas9-VP64 gene [20]. We finally obtained 2.03–2.25 × 10^12 AAV particles per milliliter in independent preparations, thus demonstrating that this construct encapsidates robustly.

We integrated an *Rluc* reporter gene, driven by the SV40 promoter, into the HEK-293T genome. One sgRNA was designed to target the SV40 promoter region, and we determined the transcriptional activation efficiency of the all-in-one AAV system using a luciferase assay (Fig. 2d). The results showed that AAV infection (MOI 50) activated *Rluc* expression up to ~ 10-fold over the sgRNA nonspecific control in HEK-293T cells, which was more efficient than the dual-AAV system (MOI 50:50) (Fig. 2e).

We then tested the ability of this system to activate expression of the endogenous human genes *VEGF* and *MALAT1*, which have been tested in our previous study [15]. For each gene, we designed three sgRNAs and assayed transcriptional activation directly by qPCR of the endogenous genes. The magnitude of *VEGF* (Fig. 2f) and *MALAT1* (Fig. 2g) activation by the dual-AAV system (MOI 50:50) was generally lower than that observed with the all-in-one AAV system (MOI 50) at these genes. In addition, we also used a wide-type SpCas9 version to construct the positive feedback loop by reducing the length of the sgRNA binding sequence (upstream of TATA box) to 14 nt. Previous studies have shown that this approach activated gene expression using an active Cas9 nuclease [21, 22]. We first examined off-target cleavages of SpCas9-VP64 using the sgRNA targeting *VEGFA* which has several off-target sites as described in a previous study [23]. Interestingly, off-target cleavages at the three tested sites (*FMN1*, *PAX6*, and *SPNS3*) were greatly reduced by SpCas9-VP64 in HEK-293T cells compared with the wild type SpCas9 (Additional file 1: Figure S6), thus indicating that VP64 domain may interfere with Cas9-mediated DNA cleavage when sgRNA does not exactly match the target.

To evaluate the DNA cleavage efficiency of the all-in-one AAV-SpCas9, a reporter reconstitution assay was used as previously described [24], in which DNA cleavage triggered the reconstitution of the active enhanced yellow fluorescent protein (*EYFP*) reporter gene from its inactive form (Additional file 1: Figure S7a). As expected, the all-in-one AAV system (MOI 50) was more effective in inducing *EYFP* expression than the dual-AAV system (MOI 50:50) (Additional file 1: Figure S7b).

To further investigate the ability of the all-in-one AAV system to mediate DNA cleavage in HEK-293T cells, we selected two endogenous genes: *DNMT1* and *MED7*. *DNMT1* encodes the major enzyme that maintains methylation patterns following DNA replication [25]. SMAD7 protein is involved in cisplatin-induced apoptosis in human cancers [26]. Using PCR and TIDE analysis, we observed that the all-in-one AAV system (MOI 50) induced much higher indel mutation rates for *DNMT1* (Additional file 1: Figure S7c) and *MED7* (Additional file 1: Figure S7d) in HEK-293T cells than did the dual-AAV system (MOI 50:50).

After DNA cleavage, we subsequently tested whether gene editing was induced when a donor sequence was co-delivered with the compact Cas9 system. Because of the large size of the SpCas9 cDNA (4.2 kb), we replaced it with the 3.2 kb *Staphylococcus aureus* Cas9 (SaCas9), a smaller enzyme that can edit or regulate the genome with efficiencies similar to those of SpCas9 [10]. Therefore, there was about 1.2 kb left on the all-in-one AAV system before reaching the 5 kb, to integrate an additional donor DNA template. The mutant EGFP (379A>T, 384C>G) reporter gene with a premature stop codon, similar to one previously described, was integrated into the HEK-293T genome [27]. EGFP expression was restored when mutation was repaired by homologous directed recombination using the wild type EGFP template (approximately 0.7 kb). We designed a mutant EGFP-specific sgRNA sequence and analyzed a proportion of EGFP-positive cells after the AAV transduction (Additional file 1: Figure S8a). The results showed that the all-in-one AAV system (MOI 50) was more efficient at mediating homologous recombination than the dual-AAV system (MOI 50:50) (Additional file 1: Figure S8b) in vitro.

### In vivo gene activation in hypercholesterolemic mice

**Fig. 3:**
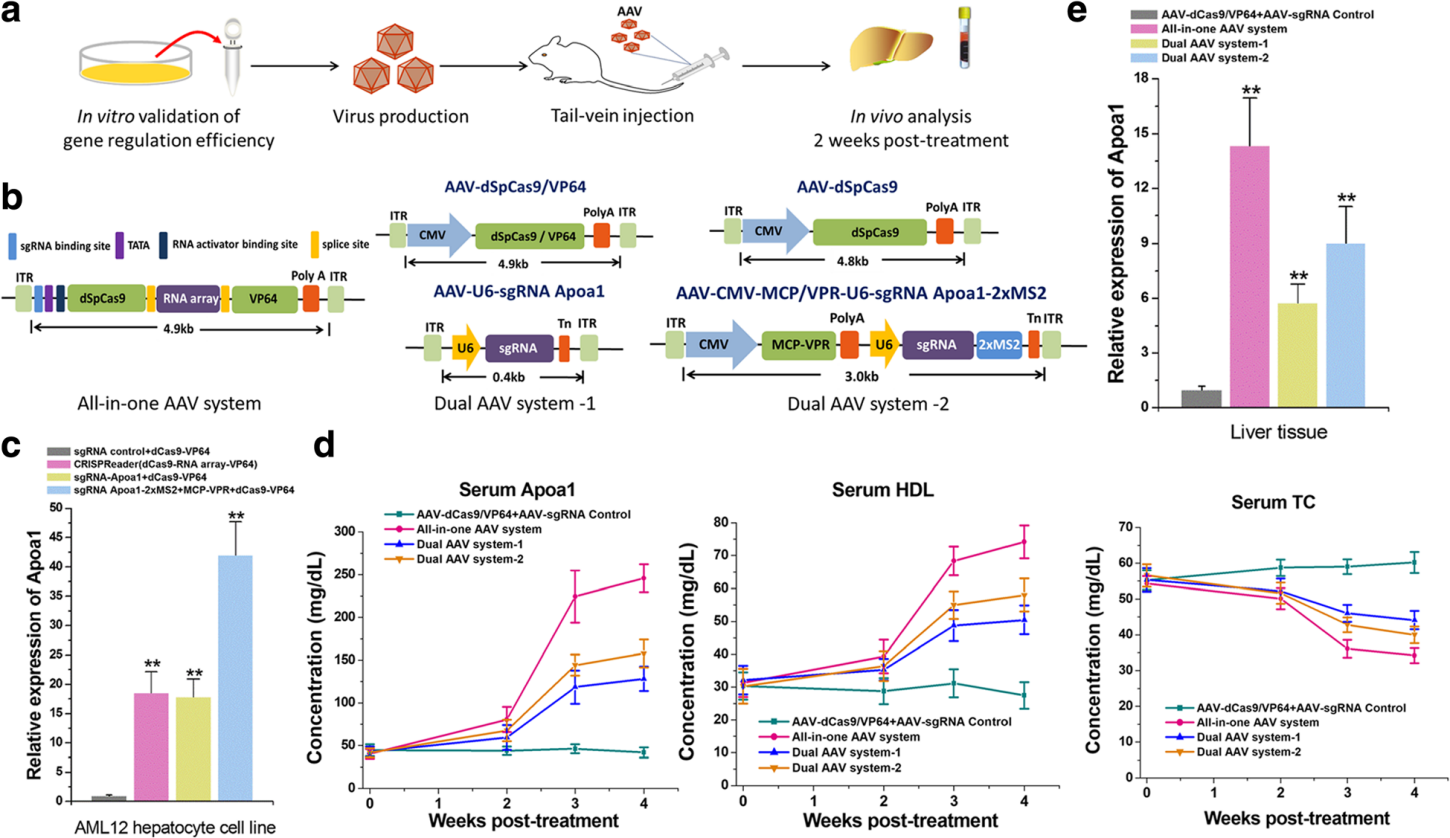
In vivo gene activation in hypercholesterolemic mice with the all-in-one AAV system. **a** Flowchart showing the key steps of AAV dCas9-mediated gene activation in the hypercholesterolemic mice model. **b** Design and construction of the all-in-one AAV-dCas9 system, as well as the two dual-AAV systems used as controls. **c** The relative mRNA levels of Apoa1 in AML12 cell line. Data are the mean ± SD from five experiments. **P < 0.01, compared with the negative control, determined with a paired, one-sided *t* test. **d** Time courses of plasma levels of Apoa1, HDL, and TC in four groups (*n* = 5 for each group) of AAV-treated hypercholesterolemic mice. Data are expressed as the mean ± SD. **e** The relative mRNA levels of Apoa1 in mice liver tissues. Data are the mean ± SD from five experiments. **P < 0.01, compared with the negative control, determined with a paired, one-sided *t* test

Finally, we evaluated the efficacy of all-in-one AAV-Cas9 system in vivo (Fig. 3a, b). For an endogenous gene target for in vivo studies, we selected *Apoa1*, a hepatocyte-specific gene targeted for activation in therapies for hypercholesterolemia induced by high-fat diets [28]. AAV-mediated *Apoa1* overexpression could reduce serum cholesterol levels with no recorded adverse side effects [28]. We first sought to show that dSpCas9-VP64 effectively activates *Apoa1* in the primary cultured AML12 hepatocyte cells. Both the CRISPReader-mediated construct (dCas9-RNA array-VP64) and the conventional dCas9-VP64/sgRNA construct were able to activate *Apoa1* expression in AML12 at 48 h after transient transfection (Fig. 3c). The strongest activation was obtained with a mixed sgRNA construct [29] containing two MS2 aptamers for recruiting the MCP-VPR (a fusion construct of the VP64, p65, and RTA domains). For targeted gene activation in vivo, we then used AAV8 with high liver tropism to generate the all-in-one AAV (dCas9-RNA array-VP64) and two different dual-AAVs, one encoding dCas9-VP64/sgRNA and the other containing dCas9/MCP-VPR/sgRNA-MS2 (Fig. 3b). We administered each AAV at the dose of 3 × 10^11^ viral genomes per vector per mouse (vg/v/m) to C57Bl/6 mice with hyperlipidemia by tail-vein injection. Delivery of dual-AAVs (3 × 10^11^ vg/v/m for each one), compared to sgRNA nonspecific control, increased Apoa1 serum levels to some extent within 4 weeks of treatment (Fig. 3d). Concomitant with increased Apoa1 serum levels, we also observed significant increases in serum levels of high-density lipoproteins (HDL) and decreases in serum levels of total cholesterol (TC) compared with sgRNA nonspecific control (Fig. 3d). Given the higher efficiency of all-in-one AAV system (3 × 10^11^ vg/v/m), serum levels of these indicators were more obviously changed (Fig. 3d). To further confirm that these effects were caused by dCas9-based gene transactivation, we detected expression of Apoa1 mRNA in the liver via qRT-PCR at 4 weeks post-treatment. In mouse livers treated with all-in-one AAV, compared with dual-AAVs and sgRNA nonspecific control, we observed much more significant transcriptional activation of the *Apoa1* gene (Fig. 3e). We also found that CRISPReader-mediated system might be a highly specific regulator in vivo, because the Apoa1 mRNA was the only transcript that was significantly activated in mouse livers at 4 weeks post-treatment (Additional file 1: Figure S9). Furthermore, we observed similar trends in lipid changes across livers isolated from these groups at 4 weeks after treatment (Additional file 1: Figure S10. Together, our results indicate that this approach resulted in the construction of a useful all-in-one AAV delivery vector for the CRISPR/Cas technology.

## Discussion

In summary, our results provide proof of principle that the CRISPReader approach can be used for multiplexed promoterless gene expression. This is an entirely new technology for regulation of exogenous transgenes with a defined molecular mechanism. It can be used to remove promoter-like gene expression regulatory elements and simplify gene cassette (Additional file 1: Figure S11a). Although the TATA box is retained, the lengthy canonical promoter regulatory sequences and translation initiation elements are completely unnecessary. The CRISPReader is simple in design and is effective without the assistance of other cofactors. This tool is highly efficient in gene expression activation and has a robust ability to control transcriptional and translational initiation. A very attractive aspect of this technique is the ability to “read” a cluster of gene ORFs without more regulatory elements, such as promoters, 5′ UTRs and IRESs. In this study, we used this approach to load the entire Cas9 system into a single AAV virus. The AAV-CRISPR-Cas9 system may be especially useful in some biomedical applications (Additional file 1: Figure S11b), although more preclinical studies are needed to assess safety and efficacy. In the future, a highly controllable minimal genome may also be constructed based on this technology for artificial life research.

## Conclusions

The CRISPReader technology described here provides a novel approach for gene regulation that simplifies gene regulatory elements and can be used in potential biomedical applications.

## Materials and methods

### Design principles for the CRISPReader

A dCas9-VP64 fusion protein consisting of the synthetic VP64 activation domain linked to the C terminus of dCas9 was constructed. The sgRNAs were designed using the online design tool “CRISPR-ERA” (http://CRISPR-ERA.stanford.edu). Along with sgRNAs, RNA activators were designed by linking a 20 nt antisense RNA to two copies of the eIF4G aptamer. The secondary structures of the RNA activators were predicted using the MFOLD program, and the RNAs which showed exposed antisense domains and maintained the natural secondary structures of eIF4G aptamers were used.

### Plasmid construction

Human codon-optimized SpCas9 fused to an NLS (Addgene plasmid # 41815; Cambridge, MA, USA) and M-SPn-Cas9-VP64 (Addgene plasmid # 48674) were used to construct natural and mutant SpCas9 expression cassette, respectively. The cDNA sequences of SaCas9 were amplified from pX602-AAV-TBG::NLS-SaCas9-NLS-HA-OLLAS-bGHpA;U6::BsaI-sgRNA (Addgene plasmid # 61593). The designed cDNA sequences for sgRNA binding regions, TATA box, RNA activator regions, MCP-VPR fusion gene, and sgRNA extended with MS2 aptamers were synthesized and inserted into corresponding vector digested with restriction endonucleases. The sequence information is shown in Additional file 2: Table S1. All vectors were transformed into One Shot TOP10 Chemically Competent *Escherichia coli* cells, and the desired expression clones were identified using polymerase chain reaction (PCR) amplification and electrophoresis, and then confirmed with Sanger sequencing.

### Cell culture and cell transfection

HEK-293T and AML12 cells were purchased from American Type Culture Collection (Manassas, VA, USA) and maintained in DMEM supplemented with 10% fetal bovine serum (Invitrogen, Carlsbad, CA, USA) in the presence of 5% CO_2_ at 37 °C in an incubator. For transient transfection experiments, cells were treated with the mixtures of plasmids using Lipofectamine 2000 Transfection Reagent (Invitrogen) according to the manufacturer’s protocols after they reached 70–80% confluency.

HEK-293T cells stably expressing *Renilla* luciferase or EGFP were obtained by transfecting cells with pcDNA3/SV40p/Rluc/Neo or pcDNA3/SV40p/EGFP/Neo and selecting positive clones with G418. In details, stable selections were carried out in 6-well plates seeded with ~ 2 × 10^5^ HEK-293T cells per well, where 2 μg of the linearized plasmids were transfected using Lipofectamine 2000 according to the manufacturer’s instructions. Cell monolayers were trypsinized 48 h after transfection and transferred into T25 flasks or 100-mm-diameter culture dishes. A mixed population of stable transfectants was selected by growth in complete medium containing 500 μg of G418/mL. These multiclonal cell lines were expanded and then verified by the luciferase reporter gene assay or PCR assay.

### Adeno-associated virus (AAV) packaging, purification, and infection

The pAAV packaging DNA construct, pHelper construct, and pAAV construct were co-transfected into HEK-293T cells with Lipofectamine2000. The culture supernatants were collected at 48 h after transfection, concentrated, and used as virus stocks for the following AAV infection experiment. The AAV titer was calculated by qPCR using 2× EvaGreen Master Mix (Syngentech).

### Luciferase assay

Relative *Renilla* luciferase activity was detected by the Dual-Luciferases Reporter Assay kit (Promega, Madison, WI, USA) using a Multimode Detector (Beckman Coulter,, Brea, CA, USA) according to the manufacturer’s instructions at 48 h post-transfection. The assays were performed in duplicate, and the experiments were repeated five times.

HEK-293T cells stably expressing the *Renilla* luciferase reporter system were seeded in 6-well plates (5 × 10^5^ /well). Forty-eight hours after transfection, the medium was removed and the cells were lysed in 500 μL of lysate buffer (Analytical Luminescence Laboratories, Dickinson, TX, USA). *Renilla* luciferase activity was measured with the *Renilla* Luciferase Reporter Assay System (Promega) according to the manufacturer’s instructions. *Renilla* luciferase activities were corrected for variation in protein concentrations of the cell extracts (Bio-Rad, Hercules, CA, USA). The assays were performed in duplicate, and the experiments were repeated three times.

### In vitro detection of GFP/RFP/YFP expression

The cells were cultured with normal growth medium, transfected with plasmids, and then examined for GFP/RFP expression using fluorescence microscopy (MicroPublisher 3.3 RTV; Olympus, Tokyo, Japan). Images were captured using the auto-exposure mode. For fluorescence measurements, the cells were trypsinized 48 h after transfection and centrifuged at 300×*g* for 7 min at 4 °C. The supernatant was removed, and the cells were resuspended in 1 × phosphate-buffered saline that did not contain calcium or magnesium. A Fortessa flow analyzer (BD Biosciences, Franklin Lakes, NJ, USA) was used for fluorescence-activated cell sorting (FACS) analyses using standard settings. GFP (or YFP) expression intensity was then measured.

### RNA extraction and real-time quantitative PCR

Tissue samples were stored in RNALater (Ambion), and total RNAs were isolated from HEK-293T cells or liver tissues using TRIzol reagent (Invitrogen, Carlsbad, CA, USA) according to the manufacturer’s protocol. The concentration and purity of total RNA were measured using UV spectrophotometric analysis at 260 nm. cDNAs were synthesized using a Revertra Ace qPCR RT Kit (Toyobo, Osaka, Japan). Real-time PCR was carried out using real-time PCR Master Mix (Toyobo). GAPDH was selected as the endogenous control. The PCR mixtures were prepared according to the manufacturer’s protocols, and amplification was performed under PCR conditions of 40 cycles of 15 s at 95 °C, 20 s at 55 °C, and 30 s at 70 °C on a ABI PRISM 7300 Fluorescent Quantitative PCR System (Applied Biosystems, Foster City, CA, USA). Primer sequences were shown in Additional file 2: Table S2. Expression fold changes were calculated using the 2^-△△ct^ method.

### Determination of NHEJ-mediated indel mutations

Cells were harvested 2 days after transfection, and the genomic DNA was extracted using the QuickExtract DNA Extraction system (Epicentre). PCR was then performed to amplify the target regions using the genomic DNA as template. The PCR products were purified using the ISOLATE II PCR and Gel Kit (Bioline) and sent for Sanger sequencing. Total NHEJ frequencies were further calculated by decomposition of the sequencing chromatogram using the TIDE, an interactive software program (https://tide-calculator.nki.nl/), as described previously [30, 31]. Depicted values were generated from TIDER analyses with *R*^2^ values > 0.9 and *P* < 0.001.

### In vivo gene activation in hypercholesterolemic mice

All experiments involving animals were housed and handled in accordance with protocols approved by the Institutional Review Board of Shenzhen University. Four-week-old C57Bl/6 mice were obtained from the Animal Center of the Academy of Sciences. Hypercholesterolemia was induced by a high-fat diet (normal stock diet containing soybean oil and an additional 1% cholesterol). Within an experiment, 20 mice aged matched by date of birth were assigned randomly to a treatment group (*n* = 5 for each group), and the tail vein was injected with AAV solution (3 × 10^11^ vg/v/m /total dose) with a 31-gauge needle. Blood samples were obtained from the retro-orbital plexus in all mice before AAV injection and every week for a total of 4 weeks after AAV treatment. Mice were euthanized 4 weeks after the AAV injection, and liver specimens were harvested and processed for *Apoa1* gene expression analysis.

### Quantification of Apoa1, HDL, and TC plasma levels

Apoa-1 levels in mouse plasma were detected with a commercially available enzyme-linked immunosorbent assay (AlerCHEK Inc., Portland, ME, USA) according to the manufacturer’s guidelines. TC and HDL levels were measured in serum via a colorimetric assay according to the manufacturer’s instructions.

### Analysis of the specificity of all-in-one AAV

Total RNA was extracted and mixed with oligo (dT) [25] Dynabeads, and mRNA was then purified with the manufacturer’s protocol (Invitrogen). The purified mRNA was first fragmented with divalent cations before library preparation. A linker was ligated to the fragmented RNA with truncated T4 RNA ligase 2 (NEB) according to the manufacturer’s instruction, and then the RNA was reverse transcribed to DNA using SuperScript® III (Thermo Scientific, Scotts Valley, CA, USA) and circularized using Circligase™ (EpiBio Madison, WI, USA). Barcodes were added by PCR using Phusion® polymerase (Thermo Scientific). The DNA library was analyzed on the Illumina HiSeq 2500 platform. Reads were processed with Cufflinks v2.1.1, and fold changes were finally calculated according to the fragments per kb of transcript per million mapped reads (FPKM) values.

### H&E staining of the livers

Liver tissues were fixed in 10% formalin and dehydrated in ethanol. Paraffin embedding, sectioning, and hematoxylin and eosin staining were performed in accordance with the manufacturer’s instructions, and then slides were imaged at × 200 magnification on a Nikon Ci-L bright field microscope. The NAFLD activity score (NAS) was calculated by an experienced pathologist. The scoring criteria were based on the criteria set by the National Institute of Health (NIH) in the USA.

### Statistical analyses

No statistical methods were used to predetermine sample size. The investigators were blinded to allocation during the experiments and outcome assessment. Statistical analyses were conducted using *t* test or analysis of variance, and *P* < 0.05 was considered statistically significant. All statistical tests were performed by SPSS statistical software for Windows, version 19.0 (SPSS, Chicago, IL, USA).

## Additional files

Additional file 1: **Figure S1.** CRISPR-based transcriptional factors drive promoterless *Rluc* gene expression. **Figure S2.** Relative activities of Rluc in HEK-293T cells transfected with the vectors containing variant mismatched RNA activators. **Figure S3.** Comparing the activation effects of dCas9-VP64/sgRNA with different numbers of binding sites. **Figure S4.** Comparing the activation effects of RNA activators with different numbers of binding sites. **Figure S5.** Comparing the translation activation efficiencies between the RNA activator and the IRES element. **Figure S6.** Comparing the DNA cleavage efficiencies between the Cas9-VP64 system and the wild type Cas9 system. **Figure S7.** Comparing the DNA cleavage efficiencies between the CRISPReader and the traditional SpCas9 system. **Figure S8.** Comparing the gene editing efficiencies between the CRISPReader and the traditional SaCas9 system. **Figure S9.** Analysis of the specificity of all-in-one AAV dCas9 system in vivo. **Figure S10.** Histopathological inspection of the mouse livers treated with AAVs. **Figure S11.** The possible applications of CRISPReader. (DOC 4458 kb)

Additional file 2: **Table S1.** The cDNA sequences of the engineered elements used in this study. **Table S2.** Primer sequences used in real-time quantitative PCR. (DOC 72 kb)

Additional file 3: Review history. (DOC 36 kb)

## Abbreviations

AAV: Adeno-associated virus
CMV: Cytomegalovirus
CRISPR: Clustered regularly interspaced short palindromic repeats
CRISPReader: (CRISPR)-Reader
*EGFP*: Enhanced green fluorescent protein
eIF4G: Eukaryotic initiation factor 4G
*EYFP*: Enhanced yellow fluorescent protein
HDL: High-density lipoproteins
HEK-293T: Human embryonic kidney 293T
IRES: Internal ribosome entry site
*Rluc*: *Renilla* luciferase
SaCas9: *Staphylococcus aureus* Cas9
sgRNA: Single guide RNA
SpCas9: *Streptococcus pyogenes* Cas9
TC: Total cholesterol
UTRs: Untranslated regions
vg/v/m: Viral genomes per vector per mouse
