# Supplementary material for: Multiplexed promoterless gene expression with CRISPReader
Source: Genome Biol. 2019 Jun 3;20:113. doi: 10.1186/s13059-019-1712-5 (PMC6545682; doi:10.1186/s13059-019-1712-5)
Supplement: Supplementary file 1 — Figure S1. CRISPR-based transcriptional factors drive promoterless Rluc gene expression. Figure S2. Relative activities of Rluc in HEK-293T cells transfected with the vectors containing variant mismatched RNA activators. Figure S3. Comparing the activation effects of dCas9-VP64/sgRNA with different numbers of binding sites. Figure S4. Comparing the activation effects of RNA activators with different numbers of binding sites. Figure S5. Comparing the translation activation efficiencies between the RNA activator and the IRES element. Figure S6. Comparing the DNA cleavage efficiencies between the Cas9-VP64 system and the wild type Cas9 system. Figure S7. Comparing the DNA cleavage efficiencies between the CRISPReader and the traditional SpCas9 system. Figure S8. Comparing the gene editing efficiencies between the CRISPReader and the traditional SaCas9 system. Figure S9. Analysis of the specificity of all-in-one AAV dCas9 system in vivo. Figure S10. Histopathological inspection of the mouse livers treated with AAVs. Figure S11. The possible applications of CRISPReader. (DOC 4458 kb) [file 13059_2019_1712_MOESM1_ESM.doc]

**
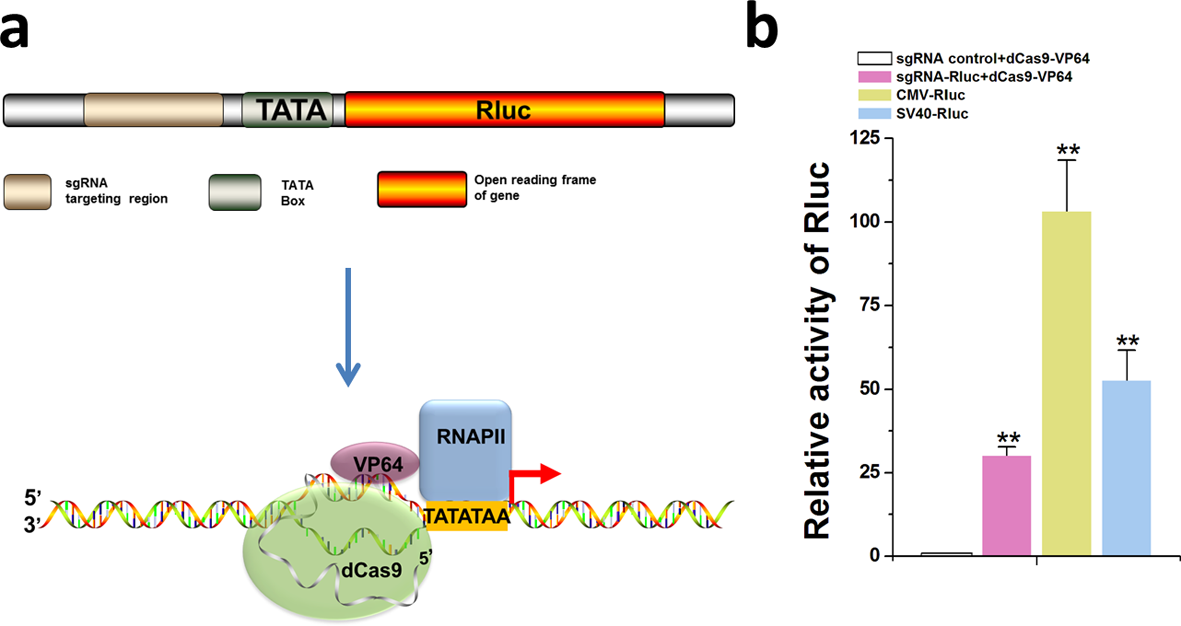
**

**Supplementary Figure1.** **CRISPR-based transcriptional factors drive promoterless *Rluc* gene expression.** (**a**) Synthetic dCas9-VP64 contained a catalytically inactive Cas9 and a VP64 transcriptional activation domain, and was guided by the sgRNA which had sequence homology to the target site upstream of the TATA box. The VP64 domain recruited RNA pol II to initiate transcription of *RLuc*. (**b**) The Results of the dual luciferase assay. An unregulated TK promoter-driven gene encoding firefly luciferase was used as a control. Reported data are the mean ± SD from five experiments. **P < 0.01, compared with the sgRNA negative control using a paired, one-sided t-test.

**
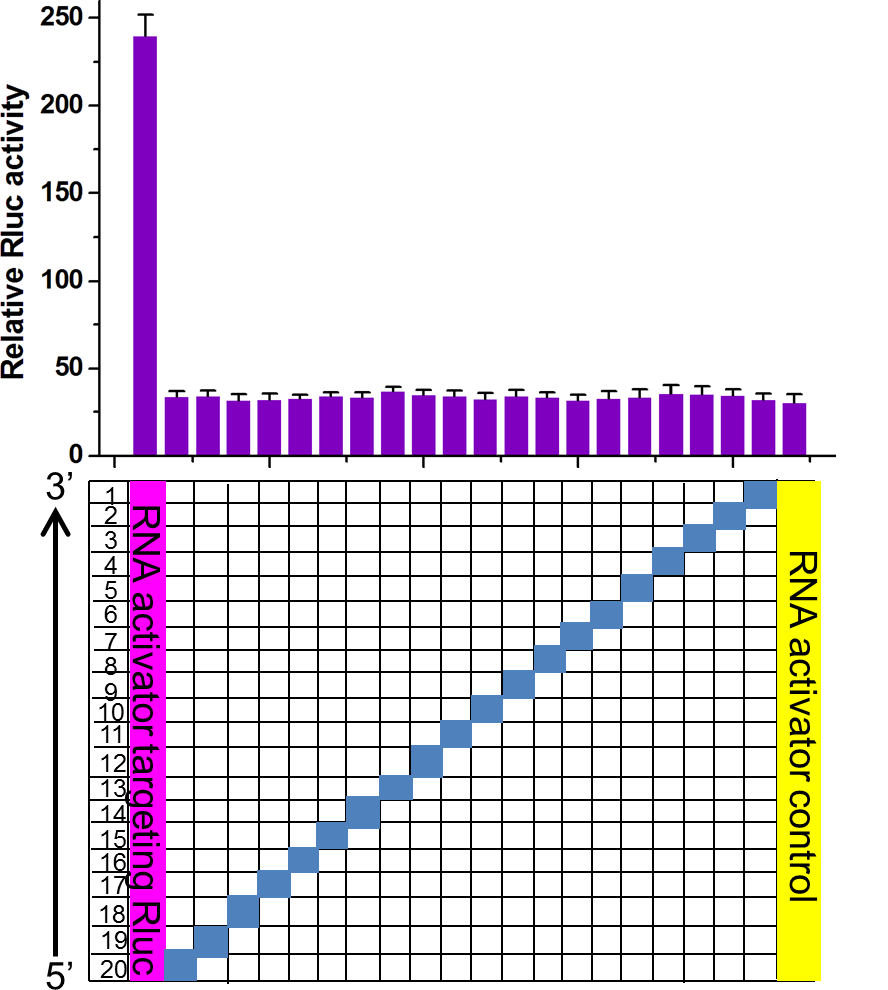
**

**Supplementary Figure 2.** **Relative activities of Rluc in HEK-293T cells transfected with the vectors containing variant mismatched RNA activators**. RNA activators bearing single mismatches were constructed. The dCas9-VP64 was transfected along with the RNA activator. As measured by luciferase assay, CRISPReader-mediated luciferase activation could be inhibited by single-nucleotide mismatches at the RNA activator-target site interface, indicating that the off-target activity of the aptamer-based RNA activator used in this study may be limited. Data are means+/−SD (n=5).


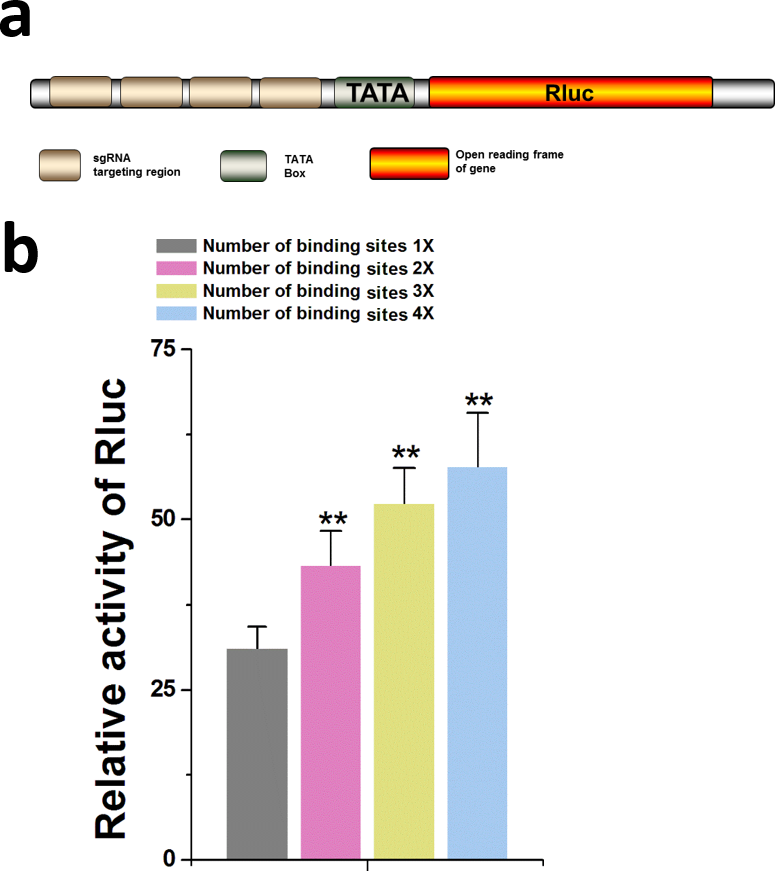


**[Supplementary](http://www.nature.com/ncomms/2015/150706/ncomms8217/full/ncomms8217.html#supplementary-information) Figure 3.** **Comparing the activation effects of dCas9-VP64/sgRNA with different numbers of binding sites.** (**a**) Various copies of sgRNA targeting region were inserted upstream of the TATA box. (**b**) As measured by luciferase assay, the activation efficiencies were compared among the luciferase constructs. An unregulated TK promoter-driven gene encoding firefly luciferase was used as a control. Data are means+/−SD (n=5). **P < 0.01, compared with the 1x dCas9-VP64/sgRNA using the paired, one-sided t-test.


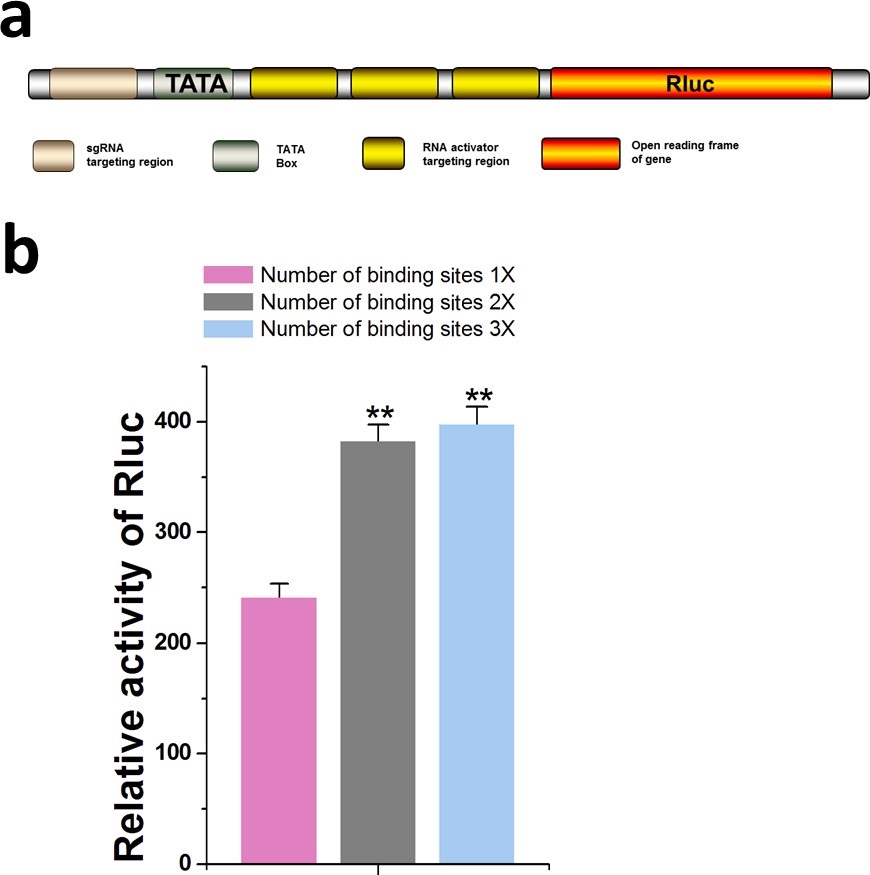


**Supplementary Figure 4.** **Comparing the activation effects of RNA activators with different numbers of binding sites.** (**a**) Various copies of RNA activator targeting region were inserted upstream of the Rluc ORF. (**b**) The dCas9-VP64 was transfected along with the RNA activator. As measured by luciferase assay, the activation efficiencies were compared among the luciferase constructs. An unregulated TK promoter-driven gene encoding firefly luciferase was used as a control. Data are means+/−SD (n=5). **P < 0.01, compared with the 1xRNA activator, one-sided t-test.


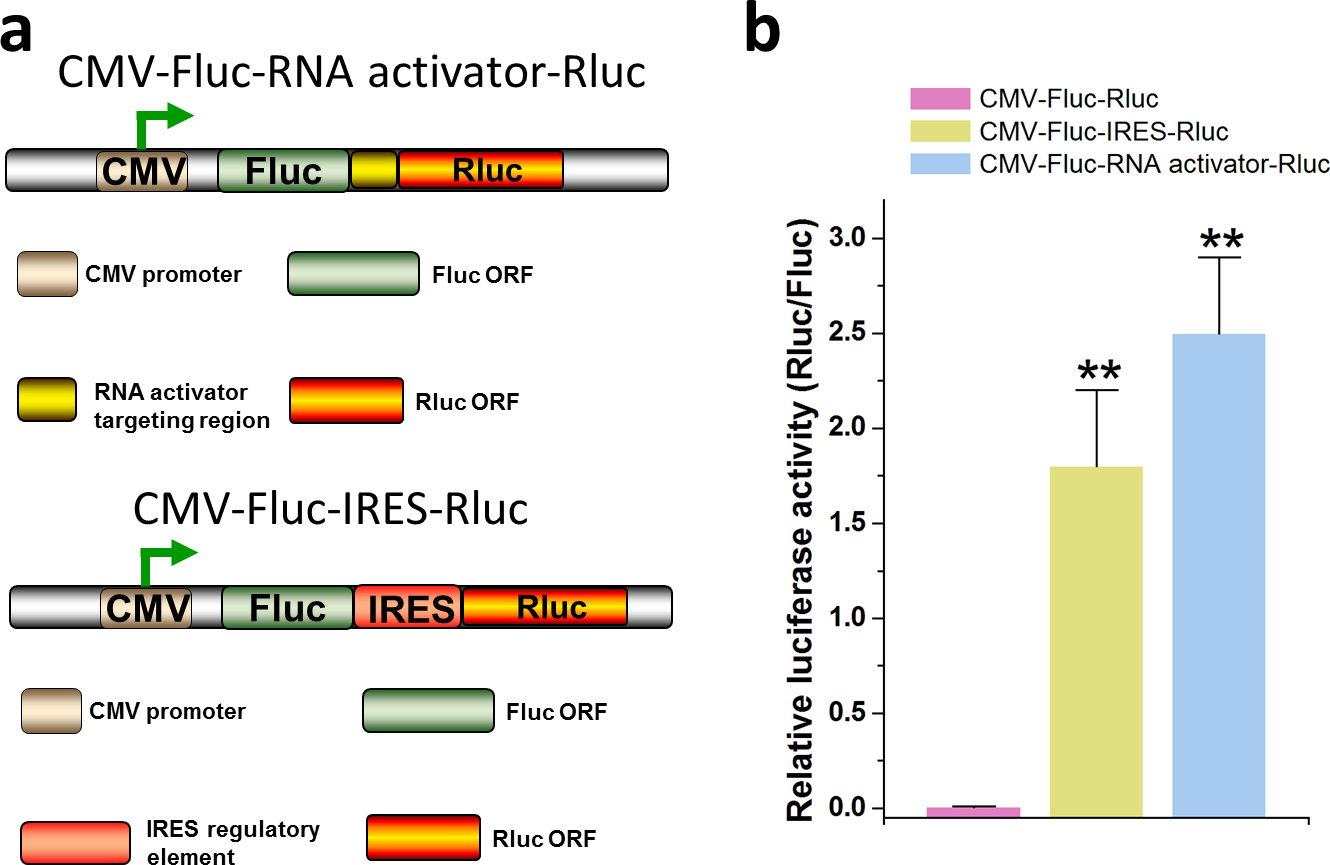


**Supplementary Figure 5. Comparing the translation activation efficiencies between the RNA-activator and the IRES element.** (**a**) The reporter plasmid consists of two ORFs. An ORF encoding Rluc was placed downstream of a primary ORF encoding Fluc. The two ORFs were separated by an RNA activator targeting region or an IRES element. For IRES-independent Rluc mRNA translation, a constitutive U6 promoter-driven gene expressing the RNA activator was also encoded on the CMV-Fluc-RNA activator-Rluc vector. (**b**) The two vectors were individually transfected into HEK-293T cells, and the activation efficiencies were compared among the constructs by luciferase assay at 48h after transfection. A CMV-Fluc-Rluc vector lacking the RNA activator targeting region was used as the negative control. Data are means+/−SD (n=5). **P < 0.01, compared with the negative control, one-sided t-test.


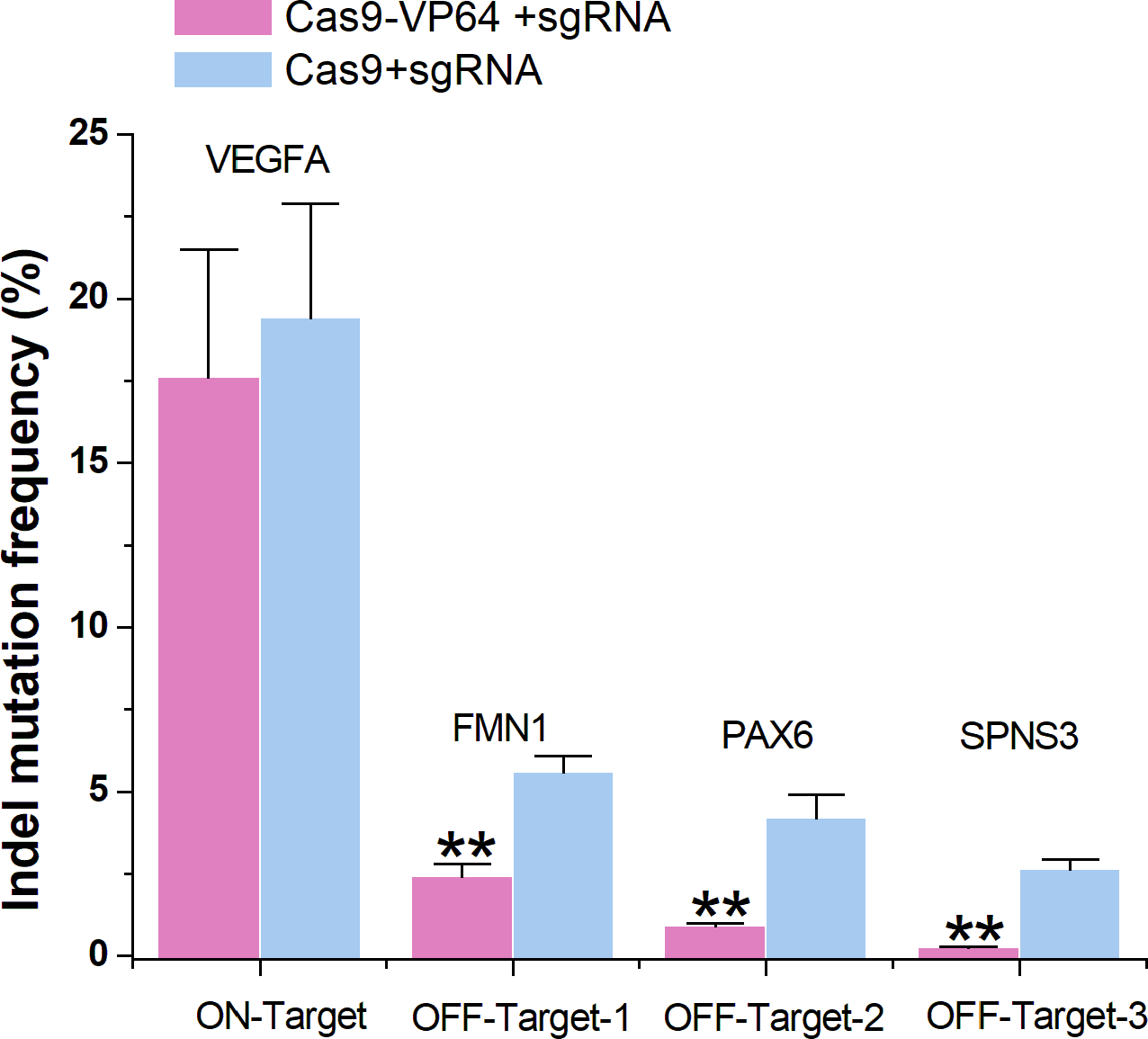


**Supplementary Figure 6. Comparing the DNA cleavage efficiencies between the Cas9-VP64 system and the wild type Cas9 system.** HEK-293T cells were transfected with plasmids encoding the SpCas9-VP64 or the wild type SpCas9. The indel frequencies for each gene were determined 2 d after transfection. **P < 0.01, compared with the control, one-sided t-test. Bars represent average indel frequencies ± SD, n = 3, as measured by tracking of indels by TIDE analysis.

**
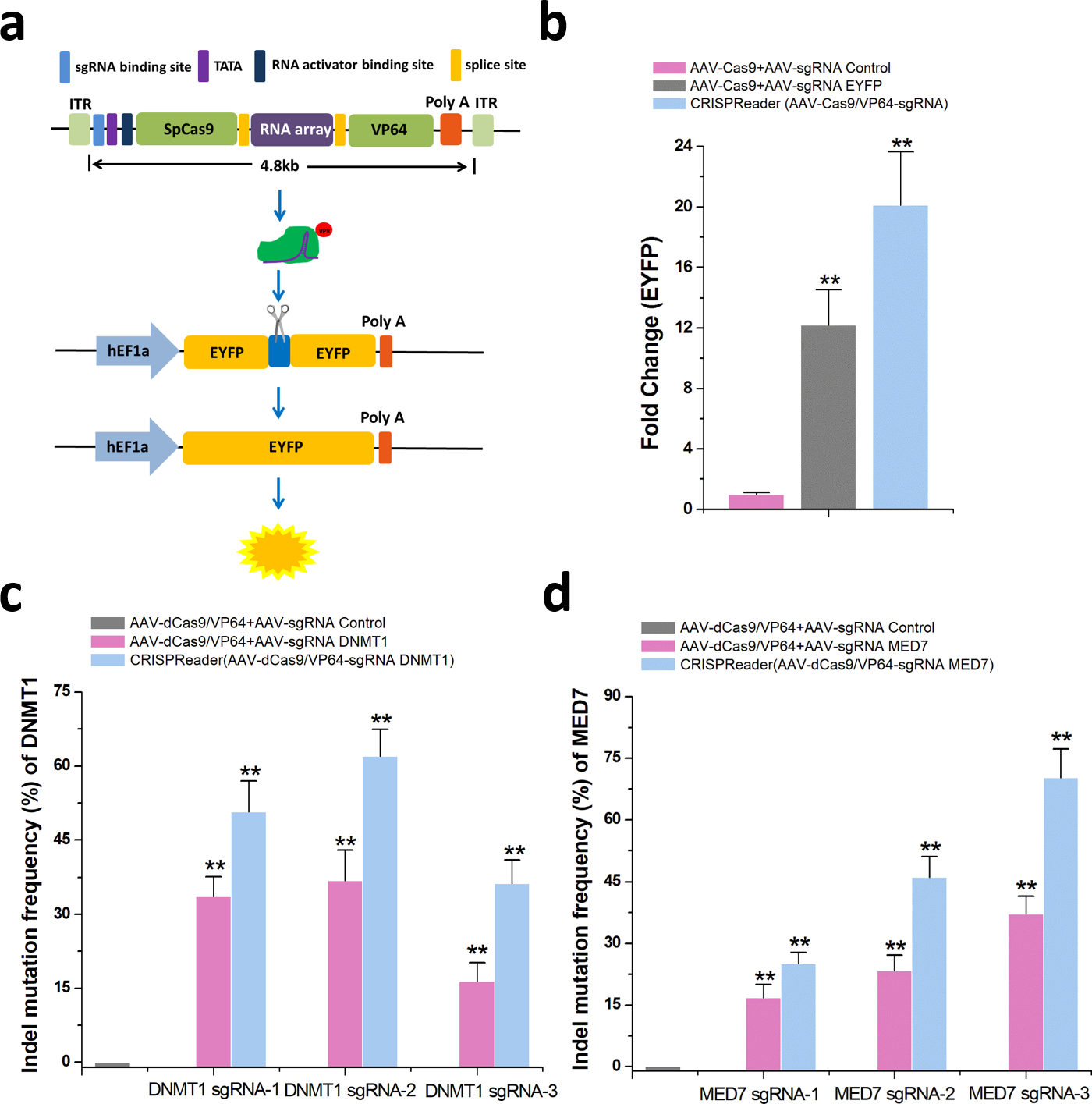
**

**Supplementary Figure 7. Comparing the DNA cleavage efficiencies between the CRISPReader and the traditional SpCas9 system.** (**a**) Diagram of reconstitution of EYFP domains for detection of DNA cleavage efficiency. A repeat sequence was inserted in the middle of EYFP reporter gene. The wide-type SpCas9 protein could cleave the EYFP repeat region, triggering the reconstitution of inactive EYFP into the full-length active EYFP. (**b**) HEK-293T cells were transfected with plasmids encoding the EYFP reporter gene. Then, AAV was added to cells 2 h after transfection. Each bar shows mean fold changes (mean ± SD; n = 5) of EYFP fluorescence measured by using FACS. **P < 0.01, compared with the sgRNA negative control, one-sided t-test. (**c** & **d**) The indel frequencies in HEK-293T cells were shown. **P < 0.01, compared with the sgRNA negative control, one-sided t-test. Bars represent average indel frequencies ± SD, n = 3, as measured by tracking of indels by TIDE analysis.


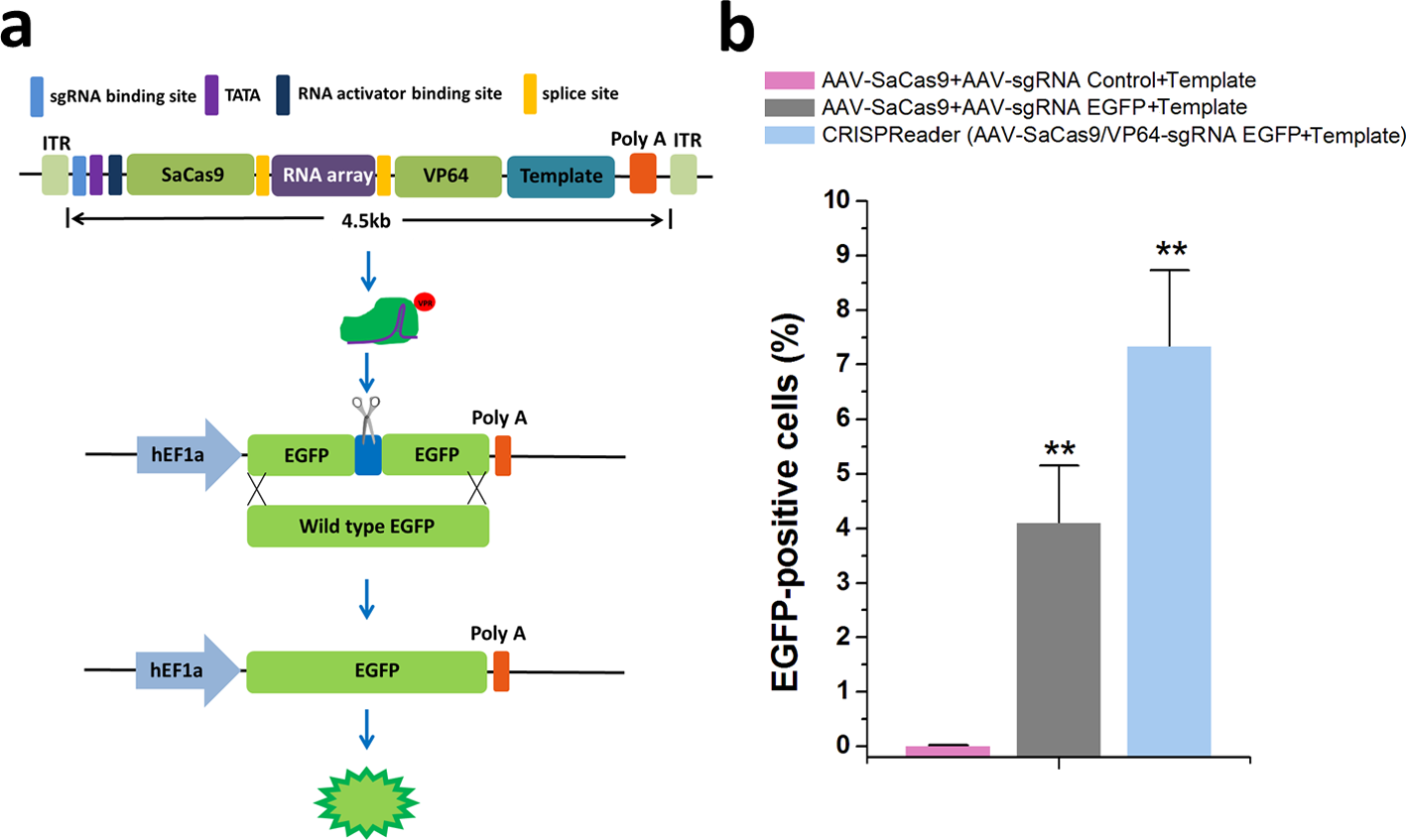


**Supplementary Figure 8. Comparing the gene editing efficiencies between the CRISPReader and the traditional SaCas9 system.** (**a**) Diagram of reconstitution of EGFP domains for detection of gene editing efficiency. A stop codon was generated in the middle of EGFP reporter gene. Homologous recombination between the introduced EGFP donor and the cleaved EGFP target results in the reconstitution of a full-length EGFP gene. (**b**) HEK-293T cells were stably transfected with plasmids encoding the mutant EGFP reporter gene. Then, AAV was added to these cells. Each bar shows the percentage of EGFP-positive cells (mean ± SD; n = 3) measured by using FACS. **P < 0.01, compared with the sgRNA nonspecific guide control, one-sided t-test.


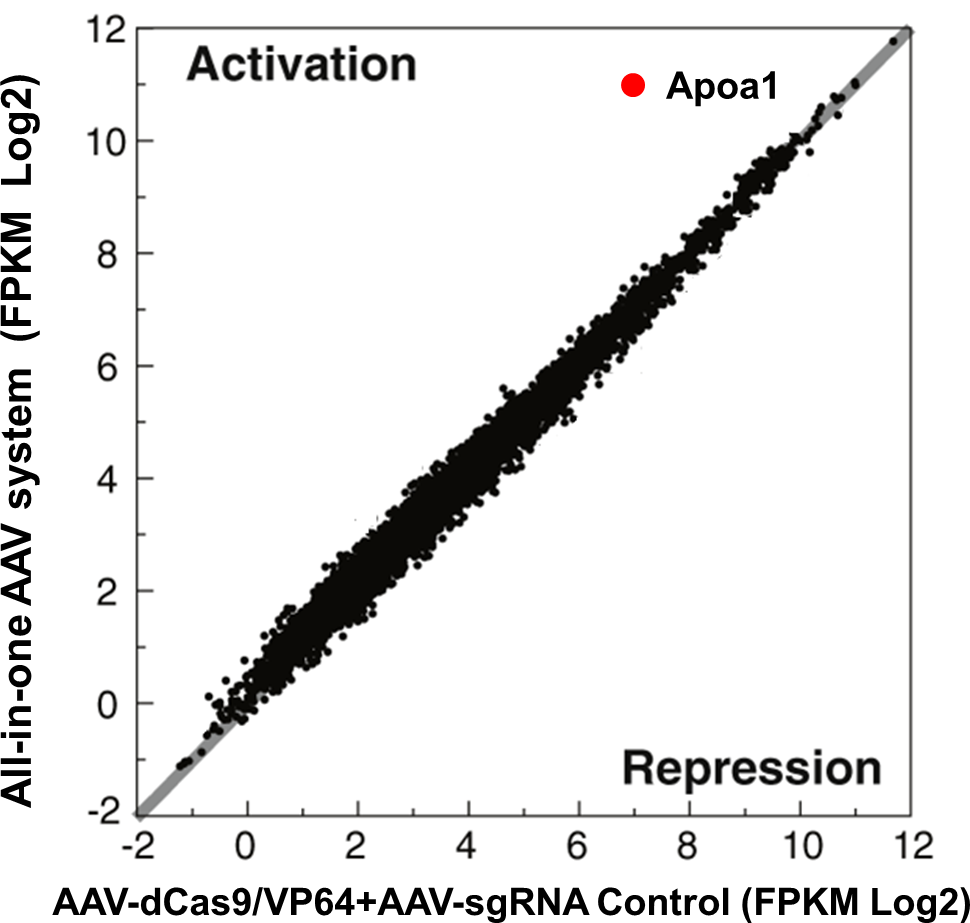


**Supplementary Figure 9. Analysis of the specificity of all-in-one AAV dCas9 system *in vivo*.** Differential expression analysis was performed comparing liver tissues from mice treated with all-in-one AAV dCas9-RNA array-VP64 system vs. dual AAV system expressing dCas9-VP64 and sgRNA control. The data point representing the Apoa1 transcript is highlighted in red. According to the calculated FPKM values, only the transcription of the Apoa1 was remarkably activated, demonstrating the high specificity of all-in-one AAV dCas9-RNA array-VP64 system. The data are representative of two independent biological replicates.


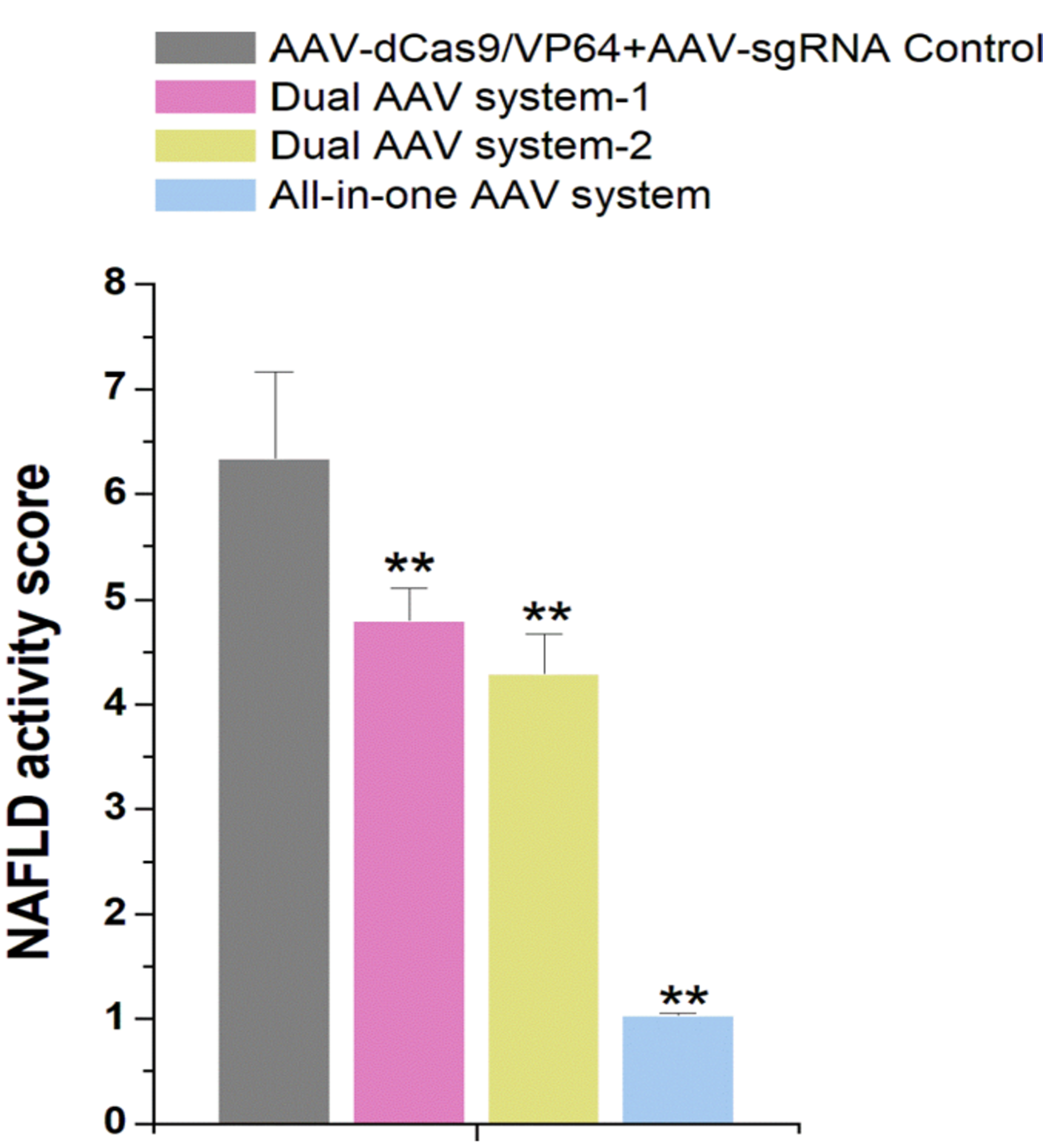


**[Supplementary](http://www.nature.com/ncomms/2015/150706/ncomms8217/full/ncomms8217.html#supplementary-information) Figure 10**. **Histopathological inspection of the mouse livers treated with AAVs.** Livers were examined with H & E staining and liver sections from mice injected with different AAVs were analyzed at 4 weeks after treatment. Hepatic histopathological scores of NAS in different groups (mean ± SD; n = 5) were shown. **P < 0.01, compared with the sgRNA negative control, one-sided t-test.


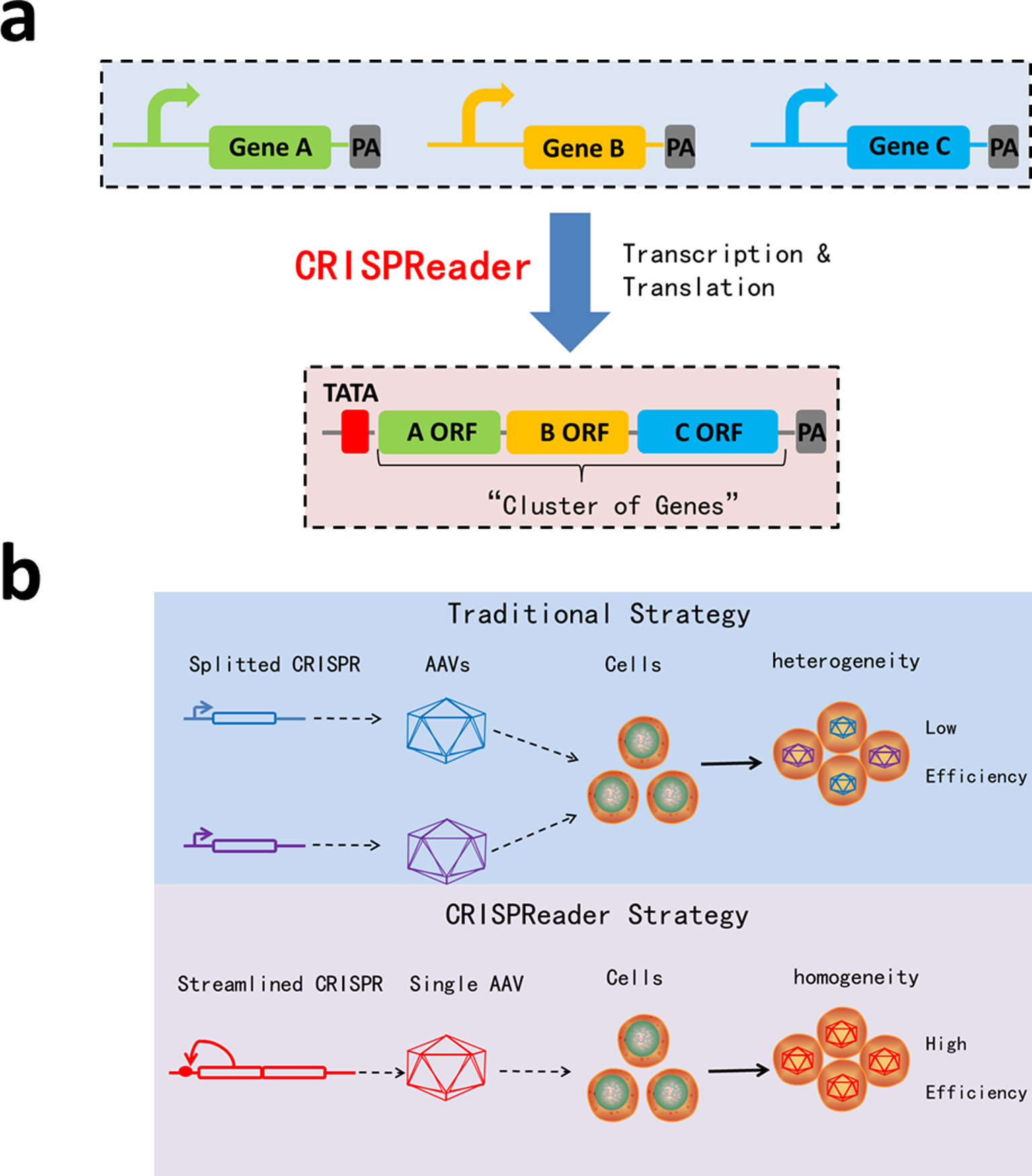


**[Supplementary](http://www.nature.com/ncomms/2015/150706/ncomms8217/full/ncomms8217.html#supplementary-information) Figure 11**. **The possible applications of CRISPReader.** (**a**) The CRISPReader could be used to reduce the size of the gene expression cassette by removing promoter-like elements to construct a compact gene-coding system. (**b**) The advantage of the all-in-one AAV-CRISPR system constructed using the CRISPReader. In traditional constructs, the Cas9 was split and packaged into two separate AAVs along with the sgRNA. This dual-AAV system reduced the delivery efficiency. CRISPReader resolved this problem by facilitating the construction of an all-in-one AAV delivery vector.
