# Supplementary material for: Multiplexed promoterless gene expression with CRISPReader
Source: Genome Biol. 2019 Jun 3;20:113. doi: 10.1186/s13059-019-1712-5 (PMC6545682; doi:10.1186/s13059-019-1712-5)
Supplement: Supplementary file 2 — Table S1. The cDNA sequences of the engineered elements used in this study. Table S2. Primer sequences used in real-time quantitative PCR. (DOC 72 kb) [file 13059_2019_1712_MOESM2_ESM.doc]

**[Supplementary](http://www.nature.com/ncomms/2015/150706/ncomms8217/full/ncomms8217.html#supplementary-information) Table 1.** The cDNA sequences of the engineered elements used in this study.

| Names | Sequences |
| --- | --- |
| sgRNA targeting region | GTGATAGAGAACGTATGTCG |
| TATA box | TATATAA |
| RNA activator binding region | CCGCGTACGAACGAGATCGC |
| RNA activator | *GCGATCTCGTTCGTACGCGG*GGGACACAATGGACGTCCGTAGAAACGCGTTAAGGTGAAAGTTTGAGGGCTCCTCATAACGGCCGACATGAGAG**CAACAACAA**GGGACACAATGGACGTCCGTAGAAACGCGTTAAGGTGAAAGTTTGAGGGCTCCTCATAACGGCCGACATGAGAG |
| RNA control | GACGTGACACGTTCGGAGAAGGGACACAATGGACGTCCGTAGAAACGCGTTAAGGTGAAAGTTTGAGGGCTCCTCATAACGGCCGACATGAGAGCAACAACAAGGGACACAATGGACGTCCGTAGAAACGCGTTAAGGTGAAAGTTTGAGGGCTCCTCATAACGGCCGACATGAGAG |
| 5' splice site | CTGGAGGCTTGCTGAAGGCTGTATGCT |
| 3' splice site | CAGGACACAAGGCCTGTTACTAGCACTCACATGGAACAAATGGC |
| 5’ processing ribozyme | CTGATGAGTCCGTGAGGACGAAATC |
| 3’ processing ribozyme | ACCGGAGTCGGGTCTGATGAGTCCGTGAGGACGAAA |
| Synthetic poly (A) | AATAAAATATCTTTATTTTCATTACATCTGTGTGTTGGTTTTTTGTGTG |
| sgRNA-SV40p (guide region) | AAGTCCCCAGGCTCCCCAGC |
| sgRNA- EYFP (guide region) | TAGATTGTGAACCCAGTGAA |
| sgRNA1-VEGF (guide region) | GAAAATTACCCATCCGCCCC |
| sgRNA2-VEGF (guide region) | GAGCTTCCCCTTCATTGCGG |
| sgRNA3-VEGF (guide region) | GGTGCTCGGACCTTGGACCG |
| sgRNA1-MALAT1 (guide region) | GAGGGACTGCGCAACCGGTG |
| sgRNA2-MALAT1 (guide region) | GCTGCGTCAGGGACAAACGC |
| sgRNA3-MALAT1 (guide region) | GCGCCGCGCAGGGATACGCG |
| sgRNA-VEGFA (guide region) | GACCCCCTCCACCCCGCCTC |
| sgRNA1-DNMT1 (guide region) | GACATCGTCGGGCAGCGAGA |
| sgRNA2-DNMT1 (guide region) | GGCGTGCGAGGTTTGGAAAG |
| sgRNA3-DNMT1 (guide region) | GCGAGGTTTGGAAAGGGGTT |
| sgRNA1-MED7 (guide region) | GATGATAGCAACAATTGTAC |
| sgRNA2-MED7 (guide region) | GGTTCTTGATAGTCCACAAT |
| sgRNA3-MED7 (guide region) | GACCTTGAGAGTCATGATGG |
| sgRNA-EGFP (guide region) | GTGAACCGCATCGAGCTGTA |
| sgRNA-Apoa1 (guide region) | GCCTAGGCCCTCACCACTCT |
| sgRNA control (guide region) | GTACGTTCTCTATCACTGATA |
| EGFP Template | GTGAGCAAGGGCGAGGAGCTGTTCACCGGGGTGGTGCCCATCCTGGTCGAGCTGGACGGCGACGTAAACGGCCACAAGTTCAGCGTGTCCGGCGAGGGCGAGGGCGATGCCAC  CTACGGCAAGCTGACCCTGAAGTTCATCTGCACCACCGGCAAGCTGCCCGTGCCCTGGCCCACCCTCGTGACCACCCTGACCTACGGCGTGCAGTGCTTCAGCCGCTACCCCG  ACCACATGAAGCAGCACGACTTCTTCAAGTCCGCCATGCCCGAAGGCTACGTCCAGGAGCGCACCATCTTCTTCAAGGACGACGGCAACTACAAGACCCGCGCCGAGGTGAAG  TTCGAGGGCGACACCCTGGTGAACCGCATCGAGCTGAAGGGCATCGACTTCAAGGAGGACGGCAACATCCTGGGGCACAAGCTGGAGTACAACTACAACAGCCACAACGTCTA  TATCATGGCCGACAAGCAGAAGAACGGCATCAAGGTGAACTTCAAGATCCGCCACAACATCGAGGACGGCAGCGTGCAGCTCGCCGACCACTACCAGCAGAACACCCCCATCG  GCGACGGCCCCGTGCTGCTGCCCGACAACCACTACCTGAGCACCCAGTCCGCCCTGAGCAAAGACCCCAACGAGAAGCGCGATCACATGGTCCTGCTGGAGTTCGTGACCGCC  GCCGGGATCACTCTCGGCATGGACGAGCTGTACAAGTAA |

Note: The cDNA sequences of RNA activator guide region (italic), eIF4G aptamer (shadowed) and spacer (bolded) were indicated.

**Supplementary Table 2**. Primer sequences used in real-time quantitative PCR.

| Names | Sequences |
| --- | --- |
| VEGF-F | ACAGACACCGCTCCTAGCCC |
| VEGF-R | CGAGAACAGCCCAGAAGTTGG |
| MALAT1-F | AAAGCAAGGTCTCCCCACAAG |
| MALAT1-R | GGTCTGTGCTAGATCAAAAGGCA |
| Apoa1-F | GACAGCGGCAGAGACTATGTGT |
| Apoa1-R | AGGAGATTCAGGTTCAGCTGTTG |
| GAPDH-F | CGCTCTCTGCTCCTCCTGTTC |
| GAPDH-R | ATCCGTTGACTCCGACCTTCAC |
