# Supplementary material for: Multiplexed promoterless gene expression with CRISPReader
Source: Genome Biol. 2019 Jun 3;20:113. doi: 10.1186/s13059-019-1712-5 (PMC6545682; doi:10.1186/s13059-019-1712-5)
Supplement: Supplementary file 3 — Review history. (DOC 36 kb) [file 13059_2019_1712_MOESM3_ESM.doc]

Review History

**First round of review**

**Comments to author and authors’ point-by-point responses to the reviewers’ comments:**

**Reviewer 1**

This paper describes a significant improvement in AAV-based CRISPR delivery systems, in combining the dual AAV constructs with a single one, by reducing the size of the CRISPR inserts. The "all-in-one" constructs considerably out-perform the dual constructs in a wide variety of tests. The results presented in this paper represent a useful addition to the potential in vivo utility of the CRISPR system.

A few suggestions for improvement of readability are listed below.

1.The TATA box is a promoter element whose activity is critical, for example, in the auto-regulatory constructs. So why are these constructs described as lacking any promoter?

*Response: Thanks very much for your suggestion. Reviewer 2 recommends using the term “promoteless”. After fully considering your valuable suggestion, we added a sentence in the Discussion Section: "Although the TATA box is retained, the lengthy canonical promoter regulatory sequences and translation initiation elements are completely unnecessary". The sentence “the CRISPR transcriptional activation system was useful for inducing transgene expression without a typical promoter” was also revised to “the CRISPR transcriptional activation system was useful for inducing transgene expression with a minimal TATA box”. We also paid attention to the sentences elsewhere in the manuscript. We strive to objectively describe the use of CRISPReader.*

2. P5 line 16:5 What is the dcas9-VP64-sgRNA control. If it is the same construct without luciferase, then it is an invalid control. Please explain

*Response: Thanks very much for your question. We are very sorry that this negative control has not been described clearly. In fact, just like the experimental group, it contains luciferase, but the sgRNA used does not target any known sequence. We have modified the statement in the original text: "After 48 h, the luciferase activity was measured, and the results indicated that the dCas9-VP64 system induced a 30-fold increase in luciferase activity compared to cells co-treated with the dCas9 -VP64-sgRNA control and the Rluc reporter (Extended Data Fig.1b)".*

3. P5, line 44: what is the negative control? Please explain in detail the nature of these controls, throughout the paper.

*Response: Thanks very much for your question. Negative control in this place refers specifically to: dCas9-VP64+sgRNA nonspecific control+ RNA activator nonspecific control. The negative controls elsewhere in the main text generally refer to: sgRNA nonspecific control. We have already indicated these information in the manuscript.*

4. P 5: line 41:replace"between the two reporter ORFS" with "between each pair of reporter ORFs"

*Response: Thanks very much for your suggestion. We have modified the original text according to your requirement.*

5. P5, line 49: what is meant by "tolerability" please explain

*Response: Thanks very much for your question. We realize that the meaning of the word "tolerability" is unclear. We replaced it with "specificity".*

6. P5, line 59: What were these reporter genes and how was their activity monitored microscopically

*Response: Thanks very much for your question. The two reporter genes expressed green fluorescent protein and red fluorescent protein, respectively. We have already indicated it in the original text. Experimental details of microscopic observation of fluorescence are described in the Methods Section: "The cells were cultured with normal growth medium, transfected with plasmids, and then examined for GFP/RFP expression using fluorescence microscopy (MicroPublisher 3.3 RTV; Olympus, Tokyo, Japan). Images were captured using the auto-exposure mode."*

7. Please explain "vg/v/m" for the non-virologist reader

*Response: Thanks very much for your suggestion. “Vg/v/m” is an acronym for “viral genomes per vector per mouse”. We have added this note to the original text.*

**Reviewer 2**

Zhan, Liu and colleagues present CRISPReader, which is a clever combination of tools that enable minimal gene constructs to be expressed despite the absence of canonical promoters and translation activation elements. The translation activation component was developed and reported previously but adds strength to the current system. The main innovations are the successful implementation of dCas9-VP64 to drive expression of ORFs preceded only by a TATA box, and the use of this minimization to fit an entire, functional dSpCas9 + guide into a single AAV vector. The initial basal expression of dSpCas9 induces a feed-forward amplification loop that drives ever-increasing expression, underlying its efficacy. In general, the experiments are well executed, controlled, and described. A few exceptions are delineated below, but these are addressable. Overall this work comprises a useful contribution to the field.

Minor comments:

The measurement of editing activity

(Supplementary Figures 6 and 7) does not meet the usual standards of the field. The Methods section says that editing sites were PCR amplified, and then individual amplicons were cloned and Sanger sequenced. Why were they not subjected to deep sequencing, as is now standard? Failing that, why was the bulk amplicon not population not subjected to deconvolution of Sanger traces using widely available online tools such as TIDE, TIDER and ICE? These methods are more quantitative and are not subject to possible cloning biases. It is not clear from the data presented how many individual Sanger clones were sequenced in each experiment, and whether the number of sequenced clones was sufficient to provide the necessary statistical power.

*Response: Thanks very much for your good suggestion. We understand that the experimental results may be affected by possible cloning biases. According to your suggestion, we re-verified the indel rate using the TIDE analysis (*<https://tide-calculator.nki.nl/>*) on the sequencing traces from the bulk population. The new results are shown in Supplementary Figures 6 and 7. Depicted values were generated from TIDER analyses with R2 values > 0.9 and P <0.001. The frequency of each indel is generally not very different from the previously calculations, and therefore the original conclusions remain valid from the new results.*

The term "Promoter Less" in the title, abstract and throughout the text does not conform to standard English usage. This needs to be corrected given the importance of the term to the paper. "Less" should be a suffix (perhaps hyphenated, perhaps not, depending on house style), not a whole separate word. Please use the term "Promoter-less" or "Promoterless" instead.

*Response: Thanks very much for your good suggestion. We used the term "Promoterless" instead.*

Page 8, top: The paragraph starting "After that, we constructed an all-in-one AAV…" is enormous, spanning two and a half pages and describing many constructs, concepts and tests. This needs to be broken up and reorganized into multiple paragraphs that are smaller and more manageable and easier for the reader to follow.

*Response: Thanks very much for your suggestion. As requested, the paragraph was broken up and reorganized into multiple paragraphs that are smaller and more manageable and easier for the reader to follow.*

Fig. 3C and 3D: the text in the key (inset) in each graph is sub-microscopic and impossible to read.

*Response: Thanks very much for your suggestion. According to your request, we have increased the font size in the figure. Also, we recommend that you read the uploaded figure directly.*

Supplementary Figure 8: Was the HDR experiment ever done with a no-guide or nonspecific guide control, as opposed to just with or without donor template? And did the donor include the full GFP ORF? If so, it is crucial to know that the GFP signal obtained depends on Cas9 cleavage - otherwise some positive signal could result from fortuitous insertion of the donor elsewhere in the genome (in an expressed, in-frame site) via illegitimate recombination.

*Response: Thanks very much for your important comments. In this experiment, the sgRNA control we mentioned does refer to the nonspecific guide control. We have re-emphasized this in the legend. We understand that the GFP ORF may have the potential to be randomly recombined into a site within the cellular genome. However, our GFP template sequence did not contain a start codon (ATG), I think this avoids the occurrence of false positive results to some extent. In addition, the nonspecific guide control group did not have significant GFP-positive cells from our results, so we speculate that the positive results observed should be mainly caused by Cas9-mediated homologous recombination.*

Supplementary Figures 3b and 4b have typographical errors: the key in each graph should say "Number of binding sites," not "Number of binding sties."

*Response: Thank you very much for pointing this out. We have made corrections in the figures.*
